# Emphysème lobaire géant chez un nourrisson

**DOI:** 10.11604/pamj.2020.37.292.26661

**Published:** 2020-12-01

**Authors:** Karima El Fakiri, Ghizlane Draiss

**Affiliations:** 1 Service de Pédiatrie A Unité de Pneumopédiatrie, Hôpital Mère-Enfant, Centre Hospitalier Universitaire Mohammed VI, Marrakech, Maroc

**Keywords:** Nourrisson, emphysème lobaire géant, lobectomie, infant, giant lobar emphysema, lobectomy

## Abstract

This study reports the case of a two-month-old male infant born to a first-degree marriage. Cesarean section was performed due to oligohydramnios and the infant received required vaccines. The initial interview conducted with parent’s highlighted dyspnea after delivery. For 14 days, he had had dry cough associated with wheezing progressing in a context of apyrexia. Clinical examination showed conscious, apyretic infant with HR 140 bpm, FR 51 cpm, SpO2 99% breathing 1 L/min with signs of respiratory distress such as substernal intercostal retraction with decreased left vescicular murmur, bilateral basithoracic sibilant sounds and crackles in the left region. Cardiovascular auscultation showed heart sounds deviated to the left side without heart murmur. The patient didn’t have abdominal distension. Chest X-ray showed distention of the left lung with lucency of the entire left lung and visible vascularization. It was associated with trans-mediastinal hernia as well as displacement of the mediastinal structures on the contralateral side and right pulmonary atelectasis. Radiological findings suggested giant emphysema in the left lobe, diaphragmatic hernia, dextrocardia and viral bronchiolitis. Angioscan showed giant emphysema in the left upper lobe. Echocardiography and abdominal ultrasound were normal. Left upper lobectomy was performed associated with anatomopathological examination, which confirmed the diagnosis of emphysema. The postoperative course was uneventful. Clinical and radiological outcomes were satisfactory after a 2-month follow-up period.

## Image en médecine

Nous rapportons le cas d’un nourrisson de sexe masculin âgé de 2 mois issu d´un mariage apparenté premier degré. L´accouchement s´est déroulé par césarienne pour oligoamnios, il est vacciné à jour. L´interrogatoire retrouve une notion de dyspnée à la naissance. Il présente depuis 14 jours une toux sèche associée à une sibilance évoluant dans un contexte d´apyrexie. A l´examen, le nourrisson était conscient, apyrétique, FC à 140 bpm, FR à 51 cpm, Spo2 à 99% sous 1L d´oxygène avec des signes de lutte respiratoire à type de tirage sus-sternal intercostal avec une diminution du murmure vésiculaire à gauche, des râles sibilants bilatéraux basithoraciques et des crépitants à gauche. L´auscultation cardiovasculaire a trouvé des bruits du cœur déviés du côté gauche sans souffle. Il n´y avait pas de distension abdominale. La radiographie thoracique a montré un poumon gauche distendu avec une hyperclarté globale de tout le poumon gauche, une vascularisation était visible. Elle est associée à une hernie transmédiastinale ainsi qu´un refoulement des structures médiastinales du coté controlatéral et une atéléctasie du poumon droit. Devant cet aspect radiologique un emphysème lobaire géant, une hernie diaphragmatique et une dextrocardie une bronchiolite virale sont à évoquer. Un angioscanner a montré un emphysème lobaire géant intéressant le lobe supérieur gauche. Une échocardiographie et une échographie abdominale étaient normales. Ainsi, Une lobectomie supérieure gauche a été réalisée avec une étude anatomopathologique confirmant l´emphysème. Les suites post opératoires étaient simples. L´évolution clinique et radiologique a été satisfaisante après un recul de 2 mois.

**Figure 1:**
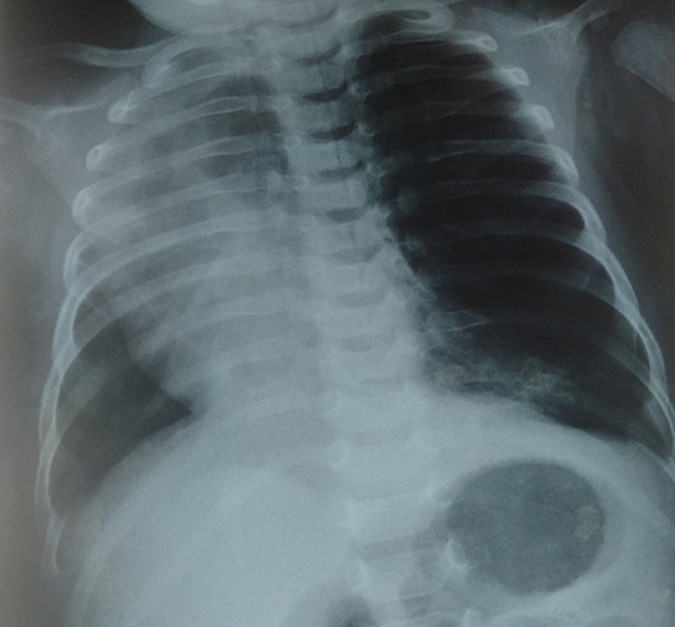
emphysème lobaire géant chez un nourrisson

